# Population aging and migration – history and UN forecasts in the EU-28 and its east and south near neighborhood – one century perspective 1950–2050

**DOI:** 10.1186/s12992-018-0348-7

**Published:** 2018-03-16

**Authors:** Mihajlo Michael Jakovljevic, Yael Netz, Sandra C. Buttigieg, Roza Adany, Ulrich Laaser, Mirjana Varjacic

**Affiliations:** 1 Global Health, Economics & Policy, Faculty of Medical Sciences, University of Kragujevac, Kragujevac, Serbia; 2 Wingate College, Israel/EGREPA - The European Group for Research into Elderly and Physical Activity, Netaniya, Israel; 3 Department of Health Services Management, Faculty of Health Sciences, University of Malta, Msida, Malta; 4 Department of Preventive Medicine, Faculty of Public Health, University of Debrecen, Debrecen, Hungary; 5 Faculty of Health Sciences, University of Bielefeld, Bielefeld, Germany; 6 Department of Fertility Pathology, Faculty of Medical Sciences, University of Kragujevac, Kragujevac, Serbia; 7 Center for Health Trends and Forecasts, Institute for Health Metrics and Evaluation, University of Washington, Seattle, USA; 8 Academy of Medical Sciences, Belgrade, Serbia

**Keywords:** Population aging, Migration, Europe, MENA, UN, WHO, Long term, Trends, History, Forecasts

## Abstract

**Background:**

There is a gap in knowledge on long term pace of population aging acceleration and related net-migration rate changes in WHO European Region and its adjacent MENA countries. We decided to compare European Union (EU-28) region with the EU Near Neighborhood Policy Region East and EU Near Neighborhood Policy Region South in terms of these two essential features of third demographic transition. One century long perspective dating back to both historical data and towards reliable future forecasts was observed.

**Methods:**

United Nation’s Department of Economic and Social Affairs estimates on indicators of population aging and migration were observed. Time horizon adopted was 1950–2050. Targeted 44 countries belong to either one of three regions named by EU diplomacy as: European Union or EU-28, EU Near Neighborhood Policy Region East (ENP East) and EU Near Neighborhood Policy Region South (ENP South).

**Results:**

European Union region currently experiences most advanced stage of demographic aging. The latter one is the ENP East region dominated by Slavic nations whose fertility decline continues since the USSR Era back in late 1980s. ENP South region dominated by Arab League nations remains rather young compared to their northern counterparts. However, as the Third Demographic Transition is inevitably coming to these societies they remain the spring of youth and positive net emigration rate. Probably the most prominent change will be the extreme fall of total fertility rate (children per woman) in ENP South countries (dominantly Arab League) from 6.72 back in 1950 to medium-scenario forecasted 2.10 in 2050. In the same time net number of migrants in the EU28 (both sexes combined) will grow from − 91,000 in 1950 to + 394,000 in 2050.

**Conclusions:**

Long term migration from Eastern Europe westwards and from MENA region northwards is historically present for many decades dating back deep into the Cold War Era. Contemporary large-scale migrations outsourcing from Arab League nations towards rich European Protestant North is probably the peak of an iceberg in long migration routes history. However, in the decades to come acceleration of aging is likely to question sustainability of such movements of people.

**Electronic supplementary material:**

The online version of this article (10.1186/s12992-018-0348-7) contains supplementary material, which is available to authorized users.

## Background

The European Commission defined the so-called EU Near Neighborhood policies and the respective regions [1]. There are two such regions. First one, named EU Near Neighborhood Southern Region that refers to the group of mostly ethnical Berber, Arab and Jewish nation states located south from Europe in MENA region of North Africa and Middle East. It consists of: Morocco, Algeria, Tunisia, Libya, Egypt, Israel, Palestinian National Authority, Jordan, Lebanon and Syrian Arab Republic. Another region named EU Near Neighborhood Eastern Region refers to the mostly Slavic Eastern European nations located in between the eastern borders of European Union and the Russian Federation. Most of these nations, with the exception of two recent withdrawals, belong to the Commonwealth of Independent States [2]. These countries are: Armenia, Azerbaijan, Georgia, Belarus, Republic of Moldova and Ukraine.

Regardless of the differences in the historical and socioeconomic legacies of these two regions with most of the EU countries, there are common public health challenges that they share. According to the huge body of evidence, population aging is a unique demographic phenomenon whose shy roots are visible in historical archives even one or two centuries ago in some European nations [3]. Nevertheless, this phenomenon has only attracted the attention of health authorities and politicians few decades ago. Since then it became a truly global change affecting all areas of life [4]. This acceleration of aging puts many nations in a very difficult perspective. The root cause of that is the pure truth that entire social support and health insurance systems of modern day welfare society were historically built up on a “demographic growth” model [5]. Instead demographic pyramids are changing its shape with younger, resource-generating levels of societies shrinking and higher upper floors of resource-consuming elderly citizens are growing and adding more weight each year [6]. Significant side of this equation is the very fact that the last year of life during suffering from terminal illness commonly costs in terms of medical care equally as the entire life time medical consumption of that citizen [7].

In recent years, the European continent has experienced the enormous demographic pressure from high net immigration rates mostly driven by refugees coming from Middle Eastern civil war affected areas [8]. These still ongoing migrations with fluctuating pace have revealed Europe’s key demographic vulnerabilities [9].

It is important to explore and compare core population aging indicators together with net-migration rates immanent to the societies within these three regions [10]. Through this analysis, we provided essentially new insights into the challenges of aging that these regions face. In addition, we also discuss the most likely direction of migration routes among them. To respond to these research questions in a long run perspective, we observed one century time horizon 1950–2050.

## Methods

United Nation’s Population Division Department of Economic and Social Affairs provides its own estimates on historical values for all the countries observed during time horizon 1950–2015 [11]. For the period 2020–2050, we complemented data with UN’s medium fertility forecasts on demographic indicators of population aging and migrations rates. These data are publicly accessible [12]. The targeted 44 countries belong to either one of three regions named by EU diplomacy as: European Union or EU-28 [13] (Austria, Belgium, Bulgaria, Croatia, Cyprus, Czech Republic, Denmark, Estonia, Finland, France, Germany, Greece, Hungary, Ireland, Italy, Latvia, Lithuania, Luxembourg, Malta, Netherlands, Poland, Portugal, Romania, Slovakia, Slovenia, Spain, Sweden and United Kingdom), EU Near Neighbourhood Policy Region East [14] (ENP East - Armenia, Azerbaijan, Georgia, Belarus, Republic of Moldova and Ukraine) and EU Near Neighbourhood Policy Region South [15] (ENP South - Morocco, Algeria, Tunisia, Libya, Egypt, Israel, Palestinian National Authority, Jordan, Lebanon and Syrian Arab Republic). Weighted average values were used to compare the three regions. In an attempt to minimize bias that could result from the large differences in population size national values, we relied on population average values. Selected indicators of aging observed were: Median age of the total population (years); Total fertility rate (children per woman); Average annual rate of population change (percentage); Old Age Dependency Ratio and Percentage of total population aged 65+ by broad age group, both sexes (per 100 total population). (Additional file 1: Table S2).

**Fig. 1:**
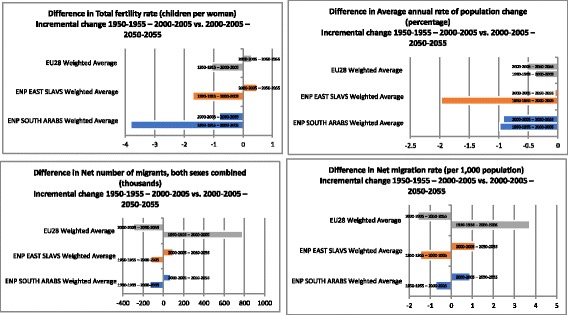
The most relevant differences in incremental change of population aging and migration indicators among the three regions (EU28/ENP East/ENP South) comparing the first half century horizon 1950–2000 with the latter one 2000–2050. Top left panel: Incremental change in total fertility rate (children per women); Top right panel: Incremental change in average annual rate of population change (percentage); Bottom left panel: Incremental change in net number of migrants, both sexes combined (thousands) and Bottom right panel: Incremental change in net migration rate (per 1000 population)

Migrations were assessed based on two key indicators whose historical availability dates back to 1950 and future forecasts are available up to 2050. These are: net number of migrants, both sexes combined (thousands) and net migration rate (per 1000 population). These data were extracted from this public registry as average national values for five-year periods: 1950–1955; 2000–2005 and 2050–2055 (Additional file 1: Table S3). Weighted average values were compared as three annual threshold values in 1950, 2000 and 2050 among the three regions to observe dynamics of changes. (Table 1) Further deeper insight into the evidence of acceleration of population aging and changing migration rates is provided by incremental change ratios comparing 1950–2000 with 2000–2050 time window (Fig. 1). Method of estimation of global and regional aggregates - Averages are weighted by population to obtain global and regional averages for income groups (World Bank classification via Atlas method) and for WHO Regions [16].

**Table 1:** Population weighted average values: key population aging and migration indicators 1950 / 2000 / 2050

		EU28 Weighted average	ENP East (predominantly Slavic Nations) Weighted average	ENP South (predominantly Arab Nations) Weighted average	WORLD
Median age of the total population (years)	1950	31.4	27.1	20.3	23.5
	2000	38.2	35.6	21.8	26.3
	2050	48.0	43.1	34.1	36.1
Total fertility rate (children per woman)	1950–1955	2.53	3.01	6.72	4.96
	2000–2005	1.47	1.31	2.91	2.62
	2050–2055	1.77	1.80	2.10	2.22
Average annual rate of population change (percentage)	1950–1955	0.72	1.41	2.62	1.77
	2000–2005	0.34	−0.57	1.63	1.24
	2050–2055	− 0.19	− 0.62	0.71	0.50
Old Age Dependency Ratio	1950	13.4	12.2	6.2	8.4
	2000	23.3	18.3	8.4	10.9
	2050	51.4	36.1	20.9	25.6
Percentage of total population aged 65+ by broad age group, both sexes (per 100 total population)	1950	8.8	7.9	3.5	5.1
	2000	15.7	12.5	5.0	6.8
	2050	29.8	22.1	13.3	16.0
Net number of migrants, both sexes combined (thousands)	1950–1955	−91	−3	−72	N/A
	2000–2005	693	− 126	− 209	N/A
	2050–2055	394	−30	−138	N/A
Net migration rate (per 1000 population)	1950–1955	−0.7	−0.1	− 0.5	N/A
	2000–2005	3.1	−1.6	− 1.2	N/A
	2050–2055	1.5	−0.5	− 0.3	N/A

## Results

This study revealed few major findings. First of all, EU median age will be growing from 31.4 back in 1950 to 48.0 in 2050; ENP East median age will change in the same time from 27.1 to 43.1 and ENP South from 20.3 to 34.1 years (Table 1). Probably the most impressive is scale of decreasing total fertility rate (children per woman) in all three regions: EU28 from 2.53 in 1950 to 1.77 in 2050; ENP East from 3.01 in 1950 to 1.80 in 2050 and most extreme change in ENP South from 6.72 back in 1950 to only 2.10 in 2050 (medium scenario UN forecast) (Table 1, Additional file 1: Table S2 and Additional file 1: Table S3; Fig. 1-top left panel). In fact, this finding should be taken seriously especially if we know that the percentage of total population aged 65+ by broad age group (both sexes combined) in EU28 will grow from 8.8% in 1950 to 29.8% in 2050, in the ENP East from 7.9% to 22.1% and in ENP South group from 3.5% to 13.3% (Table 1). Average annual rate of population change (%) in period from 1950 to 2050 will decrease in each group (EU28 from 0.72 in 1950 to − 0.19 in 2050; ENP East from 1.41 to − 0.62 and ENP South from 2.62 to 0.71 in same time period) (Table 1). Differences between incremental changes of average annual rate of population change are shown in Fig. 1- top right panel. The present results demonstrate that in EU28 group these changes will drop from − 0.38 in 1950–1955 – 2000-2005 to – 0.53 in 2000–2005 – 2050-2055 (− 0.91 for comparison period 1950–1955 – 2050-2055), meanwhile in ENP East group incremental changes are − 1.97 for 1950–1955 – 2000-2005 ratio and will be − 0.05 for 2000–2005 – 2050-2055 ratio (− 2.03 for 1950–1955 – 2050-2055 ratio) and these trend might be slightly similar in ENP South group (− 1.91 for 1950–1955 – 2050-2055 ratio) -0.99 vs. -0.92.

In the same period of time, net number of migrants in EU28 (both sexes combined) will grow from − 91,000 in 1950 to + 394,000 thousands; in the ENP East it will fall from + 3000 in 1950 to − 30,000 in 2050 while among the ENP South countries the change will be from − 72,000 in 1950 to − 138,000 in 2050 (Table 1). Incremental changes of these data for 1950–1955 – 2000-2005 and 2000–2005 – 2050-2055 periods are shown in Fig. 1-bottom left. Comparing the first half century horizon 1950–2000 with the latter one 2000–2050 in bright of net number of migrants, both sexes combined, it can be noticed that in EU28 countries this trend will not completely preserved (in the first half of horizon difference is 784 compared to − 299 in latter one). In the ENP East and ENP South countries these trends will endure various oscillations (in the first half of horizon difference is for ENP East − 122 and for ENP South − 136 compared to 96 for ENP East and 71 for ENP South in latter one). In bright of these results net migration rate (per 1000 population) will increase from − 0.7 in 1950 to 1.5 in 2050 for EU28 countries as well as in ENP South countries from − 0.5 to − 0.3, but at the same period of time, it will decrease in the ENP East from − 0.1 to − 0.5 (Table 1). Incremental changes of these data for 1950–1955 – 2000-2005 and 2000–2005 – 2050-2055 periods are shown in Fig. 1-bottom right. In the first half of horizon in EU28 countries net migration rate will increase (3.7) and it will be follow by decrease (− 1.6) in latter horizon of time. In ENP East and ENP South countries in the first period of time horizon net migration rate will decrease (− 1.5 for East and − 0.7 for South) and then in the other half it will be followed by slight increase (1.1 for East and 0.9 for South).

## Discussion

Early industrialized Asian nations such as Japan began to age rapidly in post WWII period and reached the most advanced stage of aging so far [17]. This fact led to acquisition of early unique real-life experiences related to the fiscal sustainability of national social and health support funds and long-term home care [18]. Other nations are approaching this issue with a vivid concern and difficulties [19]. Acceleration of demographic aging in leading developing nations, such as the example of BRICS will place additional challenge in front of health care reforms in majority of these economies as we approach 2025 [20]. Russian Federation is here presenting weighted majority of Commonwealth of Independent States nations in our findings. The process is spreading in the Third World countries and rapidly developing emerging markets as well [21]. Here we come to the paradox that rich Western nations such as France had 115 years to adapt since the process was rather slow there while Brazil will age to approximately the same extent in only 21 years [22].

European Union, regardless of all its inner diversity, experiences the most advanced stage of demographic aging compared with the remaining two regions. The latter one is the ENP East region dominated by Slavic nations whose fertility decline continues since the USSR Era back in late 1980s [23]. Future projections as we approach middle of the XXI century indicate accelerated aging bringing the issue to the front line of setting up national policy priorities. ENP South region is probably the most interesting one for several reasons. First is the fact that these countries lie outside geographic European boundaries. Second is major civilizational pattern differences reflected in dominating religious heritage and traditional ways of life in these dominantly Berber and Arabic nations. The third one and probably most important is the fact that these people remain rather young compared to their northern counterparts and still in earlier stage of population aging. Although the Third demographic transition is inevitably coming to these societies they remain the spring of youth and positive net emigration rates. Such long-term relationships have been present in terms of migration from Eastern Europe westwards and from MENA region northwards for many decades dating back deep into the Cold War Era [24]. Contemporary momentum of massive, large scale migrations outsourcing from MENA region going primarily towards rich European Protestant North [25] is probably the peak of an iceberg in long migration routes history [26], as was the Muslim invasion of Spain in the early 700 s CE [27] and more recently illegal migration from Senegal to European coasts such as Lampedusa, Sicily or the Canary Islands [28]. We witnessed that these movements were triggered by civil wars of Libya, Syria [29], Iraq and Arab Spring [30] unrests taking place few years ago. Complex medium-fertility scenario forecasts on aging and migration provided by the UN Population Division provide further insight into the likely events unfolding itself in future decades [31].

World total estimated migrant stock at mid-year 1990 according to the UN Population Division Department of Economic and Social Affairs used to be 152,563,212. Out of these there was almost even distribution among the Developed regions (82,38 million) and Developing regions (70,18 million) with slight domination of the first one (54%) [32]. Some 25 years later according to latest available data for 2015 global circumstance were radically different. Total world migrant stock was assessed at 243,700,236. Now the rich, developed countries outpaced developing ones in terms of migrant stock even more: 140.48 million (57,6%) against 103.28 million people.

Aforementioned superficial change hides the profound global processes taking place beneath the surface and affecting the movement of both simple and skilled labor force as economic migrants, refugees and other major streams of travelers [33]. Accelerated globalization [34] and population aging, civil wars, shift of global economic power from the West towards Asia, huge scale of urbanization in Third World regions and rise of mega cities [35], climate change and consequences of global warming [36] in the Arctic – to name just a few.

## Conclusion

Temporary demographic dividend arising from increased proportion of working age populations will present an advantage for ENP South markets. Such opportunities might be exploited by ENP South nations in the upcoming decades while in the EU28 and ENP East it has mostly foregone. In all three groups Arab MENA countries shall so far remain the youngest nations least to be affected by population aging in the short run.

Steadily but surely the Third Demographic Transition is taking its toll to modern day Middle Eastern and North African societies as well. Probably the most remarkable feature of the evolving landscape is sudden fertility fall already taking place from seven to three children per woman in Arab League ENP South on average. These profound changes will mean slow but significant release of the migratory pressure towards European Union countries as we approach the middle of XXI century.

## Additional file

Additional file 1: **Table S2.** Key aging indicators 1950 / 2000 / 2050. **Table S3.** Key Migration indicators: 1950/2000/2050. (DOCX 26 kb)

## References

[1] European External Action Service. European Neighbourhood Policy (ENP). https://eeas.europa.eu/topics/european-neighbourhood-policy-enp_en?page=2. Accessed 07 May 2017.

[2] Commonwealth of Independent States. http://www.cisstat.com/eng/cis.htm. Accessed 07 May 2017.

[3] Jakovljevic M (2015). The aging of Europe. The unexplored potential. Farmeconomia Health Econ Ther Pathw.

[4] GBD 2015 Mortality and Causes of Death Collaborators (2016). Global, regional, and national life expectancy, all-cause mortality, and cause-specific mortality for 249 causes of death, 1980–2015: a systematic analysis for the Global Burden of Disease Study 2015. Lancet.

[5] Jakovljevic M, Laaser U. Population aging from 1950 to 2010 in seventeen transitional countries in the wider region of South Eastern Europe (Original research). SEEJPH 2015. http://www.seejph.com/index.php/seejph/article/view/49/40. 10.12908/SEEJPH-2014-42.

[6] The OS. Cost of Aging: Public Finance Perspectives for Japan. In: Aging in the United States and Japan: Economic Trends. Chicago: University of Chicago Press; 1994. p. 139–74. http://www.nber.org/chapters/c8045.pdf.

[7] Kovačević A, Dragojević-Simić V, Rancić N, Jurisević M, Gutzwiller FS, Gutzwiller F (2015). End-of-life costs of medical care for advanced stage cancer patients. Vojnosanit Pregl.

[8] Klingseis SJ (2016). Syrian refugees: are they a non-traditional threat to water supplies in Lebanon and Jordan? [thesis].

[9] Hamood S (2008). EU-Libya cooperation on migration: a raw deal for refugees and migrants?. J Refug Stud.

[10] Hugo G (2011). Future demographic change and its interactions with migration and climate change. Glob Environ Change.

[11] United Nations. United Nations Population Division - Department of Economic and Social Affairs. http://www.un.org/en/development/desa/population/. Accessed 07 May 2017.

[12] United Nations. United Nations Population Division - Department of Economic and Social Affairs. World Population Prospects: 2015 Revision. http://www.un.org/en/development/desa/population/events/other/10/index.shtml. Accessed 07 May 2017.

[13] European Union. Countries. https://europa.eu/european-union/about-eu/countries_en. Accessed 07 May 2017.

[14] European External Action Service. The Eastern Partnership. https://eeas.europa.eu/headquarters/headquarters-homepage/3768/eastern-partnership_en. Accessed 07 May 2017.

[15] European Commission. Southern Neighbourhood. https://ec.europa.eu/neighbourhood-enlargement/neighbourhood/southern-neighbourhood_en. Accessed 07 May 2017.

[16] World Health Organization. World Health Statistics 2014. Indicator Compendium. World Health Organization. http://www.who.int/gho/publications/world_health_statistics/whs2014_indicatorcompendium.pdf. Accessed 07 May 2017.

[17] Ogura S, Tachibanaki T, Wise DA. Aging Issues in the United States and Japan: University of Chicago Press; 2007. p. 421. http://press.uchicago.edu/ucp/books/book/chicago/A/bo3628236.html

[18] Ogura S, Jakovljevic M (2014). Health financing constrained by population aging - an opportunity to learn from Japanese experience. Serbian J Exp Clin Res.

[19] GBD 2015 SDG Collaborators (2016). Measuring the health-related Sustainable Development Goals in 188 countries: a baseline analysis from the Global Burden of Disease Study 2015. Lancet.

[20] Jakovljevic M, Potapchik E, Popovich L, Barik D, Getzen TE. Evolving health expenditure landscape of the BRICS nations and projections to 2025. Health Econ. 2016; 10.1002/hec.3406. 10.1002/hec.3406 27683202

[21] Jakovljevic M, Groot W, Souliotis K (2016). Editorial: health care financing and affordability in the emerging global markets. Front Public Health.

[22] The WHO Centre for Health Development in Kobe, Japan The Wisdom Years Ageing Societies http://wisdom.unu.edu/en/ageing-societies/. Accessed 07 May 2017.

[23] Zakharov S, Ivanova E. Fertility Decline and Recent Changes in Russia: On the Threshold of the Second Demographic Transition. In: RAND conference proceedings. Santa Monica, California; 1996. p. 36–83. https://www.rand.org/pubs/conf_proceedings/CF124/CF124.chap2.html.

[24] Fassmann H, Munz R (1994). European east-west migration, 1945-1992. Int Migr Rev.

[25] Lewis H, Dwyer P, Hodkinson S, Waite L (2015). Hyper-precarious lives: migrants, work and forced labour in the global north. Prog Hum Geogr.

[26] De Haas H (2006). Trans-Saharan migration to North Africa and the EU: historical roots and current trends. Migration Policy Institute.

[27] Hendrickson J. Andalusia. The Oxford Encyclopedia of the Islamic World http://www.oxfordislamicstudies.com/article/opr/t236/e1129. Accessed 07 May 2017.

[28] Mbaye LM (2014). “Barcelona or die”: understanding illegal migration from Senegal. IZA J Migr.

[29] Yazgan P, Utku DE, Sirkeci I (2015). Syrian crisis and migration. Migr Lett.

[30] Fargues P, Fandrich C. Migration after the Arab Spring. 2012 (Migration Policy Centre Research Report). Report No.: 2012/09. http://cadmus.eui.eu//handle/1814/23504. Accessed 07 May 2017.

[31] UN Population Division Department of Economic and Social Affairs. Profiles of Aging 2015. https://esa.un.org/unpd/popdev/Profilesofageing2015/index.html. Accessed 07 May 2017.

[32] United Nations, Department of Economic and Social Affairs. Trends in International Migrant Stock: Migrants by Destination and Origin (United Nations database, POP/DB/MIG/Stock/Rev.2015). 2015. http://www.un.org/en/development/desa/population/migration/index.shtml. Accessed 07 May 2017.

[33] Castles S, de Haas H, Miller MJ. The Age of Migration: International Population Movements in the Modern World: Palgrave Macmillan; 2013. p. 421. https://www.macmillanihe.com/resources/sample-chapters/9780230355767_sample.pdf.

[34] Trask B. Globalization and families: accelerated systemic social change: Springer Science & Business Media; 2009. p. 229. http://www.springer.com/gp/book/9780387882840.

[35] Cohen B (2006). Urbanization in developing countries: current trends, future projections, and key challenges for sustainability. Technol Soc.

[36] McMichael AJ, Woodruff RE, Hales S (2006). Climate change and human health: present and future risks. Lancet.

