# Supplementary material for: Population aging and migration – history and UN forecasts in the EU-28 and its east and south near neighborhood – one century perspective 1950–2050
Source: Global Health. 2018 Mar 16;14:30. doi: 10.1186/s12992-018-0348-7 (PMC5857107; doi:10.1186/s12992-018-0348-7)
Supplement: Supplementary file 1 — Table S2. Key aging indicators 1950 / 2000 / 2050. Table S3. Key Migration indicators: 1950/2000/2050. (DOCX 26 kb) [file 12992_2018_348_MOESM1_ESM.docx]

**Table 2.** Key aging indicators 1950 / 2000 / 2050

| Country | Median age of the total population (years) | | | Total fertility rate (children per woman) | | | Average annual rate of population change (percentage) | | | | | Old Age Dependency Ratio | | | Percentage of total population aged 65+ by broad age group, both sexes (per 100 total population) | | | | |
| --- | --- | --- | --- | --- | --- | --- | --- | --- | --- | --- | --- | --- | --- | --- | --- | --- | --- | --- | --- |
|  | 1950 | 2000 | 2050 | 1950-1955 | 2000-2005 | 2050-2055 | 1950-1955 | | | 2000-2005 | 2050-2055 | 1950 | 2000 | 2050 | 1950 | | | 2000 | 2050 |
| **World** | **23.5** | **26.3** | **36.1** | **4.96** | **2.62** | **2.22** | **1.77** | | | **1.24** | **0.50** | **8.4** | **10.9** | **25.6** | **5.1** | | | **6.8** | **16.0** |
| Algeria | 19.4 | 21.7 | 37.1 | 7.28 | 2.38 | 1.93 | 2.05 | | | 1.29 | 0.55 | 6.3 | 7.1 | 26.8 | 3.5 | | | 4.3 | 16.9 |
| Morocco | 19.5 | 22.7 | 38.6 | 6.61 | 2.52 | 1.85 | 3.12 | | | 0.97 | 0.20 | 5.1 | 8.6 | 27.1 | 2.9 | | | 5.2 | 17.3 |
| Egypt | 20.8 | 21.3 | 31.0 | 6.62 | 3.15 | 2.30 | 2.48 | | | 1.85 | 0.95 | 5.2 | 8.9 | 16.3 | 3.1 | | | 5.1 | 10.4 |
| Libya | 21.0 | 22.5 | 38.4 | 7.14 | 2.75 | 1.76 | 2.04 | | | 1.67 | 0.23 | 9.3 | 6.0 | 24.7 | 5.2 | | | 3.8 | 16.1 |
| Tunisia | 20.3 | 25.1 | 40.4 | 6.65 | 2.04 | 1.82 | 1.79 | | | 0.82 | 0.10 | 8.0 | 10.6 | 31.1 | 4.5 | | | 6.7 | 19.6 |
| Israel | 25.5 | 28.0 | 35.2 | 4.28 | 2.91 | 2.31 | 6.24 | | | 1.87 | 0.96 | 6.1 | 16.2 | 29.0 | 3.9 | | | 10.0 | 17.4 |
| Jordan | 17.2 | 19.5 | 32.4 | 7.38 | 3.85 | 2.09 | 7.27 | | | 2.24 | 0.84 | 9.8 | 5.3 | 17.6 | 4.8 | | | 3.1 | 11.4 |
| Lebanon | 23.2 | 26.3 | 46.8 | 5.74 | 2.01 | 1.73 | 2.76 | | | 4.18 | 0.08 | 12.4 | 11.1 | 37.4 | 7.3 | | | 7.1 | 23.3 |
| Syrian Arab Republic | 20.3 | 18.8 | 33.7 | 7.23 | 3.67 | 1.87 | 2.78 | | | 2.07 | 0.64 | 7.9 | 6.0 | 17.2 | 4.5 | | | 3.4 | 11.5 |
| Palestinian National Authority | 17.3 | 16.0 | 27.4 | 7.38 | 5.03 | 2.59 | 1.16 | | | 2.09 | 1.48 | 9.7 | 4.6 | 10.8 | 4.8 | | | 2.3 | 6.9 |
| **ENP South Arabs Weighted average** | **20.3** | **21.8** | **34.1** | **6.72** | **2.91** | **2.10** | **2.62** | | | **1.63** | **0.71** | **6.2** | **8.4** | **20.9** | **3.5** | | | **5.0** | **13.3** |
|  | |  | | | | | | | | | | | | | | | | | |
| Armenia | 22.4 | 30.3 | 46.9 | 4.49 | 1.72 | 1.66 | 2.89 | | -0.40 | | -0.67 | 14.3 | 15.6 | 39.2 | 8.3 | | 10.0 | | 24.1 |
| Azerbaijan | 22.8 | 25.6 | 38.6 | 5.49 | 2.00 | 1.92 | 2.78 | | 1.07 | | -0.17 | 11.3 | 8.8 | 26.3 | 6.9 | | 5.6 | | 16.9 |
| Georgia | 27.3 | 34.4 | 45.0 | 3.00 | 1.58 | 1.86 | 1.70 | | -1.17 | | -0.66 | 16.0 | 19.1 | 42.8 | 10.1 | | 12.5 | | 25.3 |
| Belarus | 27.2 | 36.5 | 42.2 | 2.61 | 1.26 | 1.83 | 0.15 | | -0.64 | | -0.46 | 13.2 | 19.8 | 35.6 | 8.6 | | 13.5 | | 21.8 |
| Republic of Moldova | 26.6 | 31.3 | 49.3 | 3.50 | 1.24 | 1.56 | 2.31 | | -0.21 | | -1.04 | 12.0 | 14.0 | 35.5 | 7.7 | | 9.4 | | 22.9 |
| Ukraine | 27.6 | 37.9 | 43.7 | 2.81 | 1.15 | 1.78 | 1.41 | | -0.82 | | -0.75 | 11.7 | 20.0 | 38.4 | 7.6 | | 13.8 | | 23.3 |
| **ENP East Slavs Weighted average** | **27.1** | **35.6** | **43.1** | **3.01** | **1.31** | **1.80** | **1.41** | | **-0.57** | | **-0.62** | **12.2** | **18.3** | **36.1** | **7.9** | | **12.5** | | **22.1** |
|  | |  | | | | | | | | | | | | | | | | | |
| Austria | 35.7 | 38.2 | 49.7 | 2.10 | 1.38 | 1.75 | 0.04 | 0.45 | | | -0.13 | 15.6 | 22.6 | 54.8 | 10.4 | 15.3 | | | 30.6 |
| Belgium | 35.5 | 39.1 | 44.6 | 2.34 | 1.68 | 1.88 | 0.55 | 0.56 | | | 0.11 | 16.2 | 25.8 | 46.7 | 11.0 | 16.9 | | | 26.7 |
| Bulgaria | 27.3 | 39.7 | 47.8 | 2.53 | 1.24 | 1.83 | 0.79 | -0.81 | | | -1.02 | 10.1 | 24.5 | 50.5 | 6.7 | 16.6 | | | 28.6 |
| Croatia | 27.9 | 38.9 | 49.6 | 2.73 | 1.41 | 1.66 | 0.91 | -0.23 | | | -0.63 | 12.1 | 23.2 | 52.6 | 7.9 | 15.6 | | | 29.9 |
| Cyprus | 23.7 | 31.8 | 47.5 | 3.71 | 1.59 | 1.63 | 1.41 | 1.81 | | | 0.20 | 10.1 | 15.2 | 43.4 | 6.0 | 10.2 | | | 26.2 |
| Czech Republic | 32.5 | 37.4 | 48.1 | 2.74 | 1.19 | 1.80 | 0.99 | -0.06 | | | -0.26 | 12.4 | 19.7 | 54.6 | 8.4 | 13.7 | | | 30.2 |
| Denmark | 31.7 | 38.4 | 44.1 | 2.55 | 1.76 | 1.85 | 0.79 | 0.30 | | | 0.20 | 14.0 | 22.3 | 40.9 | 9.0 | 14.9 | | | 24.3 |
| Estonia | 29.9 | 38.0 | 45.9 | 2.06 | 1.39 | 1.83 | 1.03 | -0.63 | | | -0.50 | 16.6 | 22.3 | 48.2 | 10.6 | 15.0 | | | 27.5 |
| Finland | 27.8 | 39.4 | 45.1 | 3.00 | 1.75 | 1.83 | 1.10 | 0.27 | | | 0.05 | 10.4 | 22.3 | 45.7 | 6.6 | 14.9 | | | 26.5 |
| France | 34.7 | 37.8 | 43.9 | 2.75 | 1.88 | 1.96 | 0.77 | 0.62 | | | 0.13 | 17.3 | 24.7 | 46.3 | 11.4 | 16.1 | | | 26.3 |
| Germany | 35.3 | 40.1 | 51.4 | 2.13 | 1.35 | 1.64 | 0.43 | -0.16 | | | -0.43 | 14.5 | 23.7 | 58.6 | 9.7 | 16.2 | | | 32.3 |
| Greece | 26.0 | 38.4 | 52.3 | 2.29 | 1.28 | 1.59 | 0.95 | 0.21 | | | -0.56 | 10.5 | 24.9 | 66.0 | 6.8 | 16.9 | | | 34.8 |
| Hungary | 30.1 | 38.6 | 47.8 | 2.69 | 1.3 | 1.66 | 1.03 | -0.25 | | | -0.54 | 11.6 | 22.3 | 47.0 | 7.8 | 15.1 | | | 27.6 |
| Ireland | 30.0 | 31.8 | 42.6 | 3.42 | 1.97 | 1.95 | -0.10 | 1.80 | | | 0.31 | 18.2 | 15.5 | 45.6 | 11.0 | 10.5 | | | 25.8 |
| Italy | 28.6 | 40.4 | 51.7 | 2.36 | 1.3 | 1.73 | 0.74 | 0.52 | | | -0.36 | 12.4 | 26.7 | 67.6 | 8.1 | 18.1 | | | 35.1 |
| Latvia | 30.5 | 37.9 | 45.3 | 2.00 | 1.29 | 1.78 | 0.67 | -1.25 | | | -0.58 | 17.6 | 22.4 | 42.5 | 11.2 | 15.0 | | | 25.3 |
| Lithuania | 27.8 | 36.0 | 44.3 | 2.71 | 1.28 | 1.82 | 0.48 | -0.84 | | | -0.46 | 14.9 | 20.9 | 37.8 | 9.4 | 13.9 | | | 23.1 |
| Luxembourg | 35.0 | 37.3 | 42.7 | 1.98 | 1.65 | 1.77 | 0.62 | 0.97 | | | 0.68 | 13.9 | 21.0 | 38.7 | 9.8 | 14.1 | | | 23.4 |
| Malta | 23.7 | 35.9 | 50.2 | 4.14 | 1.47 | 1.72 | 0.12 | 0.51 | | | -0.29 | 9.7 | 17.2 | 52.0 | 5.8 | 11.6 | | | 29.7 |
| Netherlands | 28.0 | 37.5 | 46.2 | 3.05 | 1.73 | 1.84 | 1.26 | 0.54 | | | -0.10 | 12.2 | 20.0 | 47.9 | 7.7 | 13.6 | | | 27.5 |
| Poland | 25.8 | 35.4 | 51.8 | 3.63 | 1.26 | 1.60 | 1.88 | -0.01 | | | -0.69 | 8.0 | 17.9 | 55.8 | 5.2 | 12.2 | | | 31.4 |
| Portugal | 26.1 | 37.9 | 52.5 | 3.10 | 1.45 | 1.56 | 0.57 | 0.39 | | | -0.49 | 11.0 | 24.0 | 66.4 | 7.0 | 16.3 | | | 35.2 |
| Romania | 26.3 | 34.9 | 48.1 | 3.06 | 1.32 | 1.74 | 1.48 | -0.66 | | | -0.86 | 8.7 | 20.1 | 51.8 | 5.7 | 13.6 | | | 29.2 |
| Slovakia | 27.0 | 33.9 | 49.0 | 3.50 | 1.22 | 1.70 | 2.08 | 0.00 | | | -0.52 | 10.3 | 16.5 | 49.5 | 6.6 | 11.4 | | | 28.6 |
| Slovenia | 27.7 | 38.1 | 49.3 | 2.68 | 1.21 | 1.84 | 0.72 | 0.08 | | | -0.38 | 10.7 | 20.1 | 61.2 | 7.0 | 14.1 | | | 32.5 |
| Spain | 27.5 | 37.6 | 51.8 | 2.53 | 1.29 | 1.63 | 0.72 | 1.47 | | | -0.34 | 10.9 | 24.3 | 69.5 | 7.2 | 16.6 | | | 35.8 |
| Sweden | 34.2 | 39.4 | 42.0 | 2.24 | 1.67 | 1.94 | 0.70 | 0.35 | | | 0.50 | 15.3 | 26.8 | 40.5 | 10.2 | 17.3 | | | 23.8 |
| United Kingdom | 34.9 | 37.6 | 43.3 | 2.18 | 1.66 | 1.89 | 0.20 | 0.45 | | | 0.27 | 16.2 | 24.3 | 42.2 | 10.8 | 15.8 | | | 24.7 |
| **EU28 Weighted average** | **31.4** | **38.2** | **48.0** | **2.53** | **1.47** | **1.77** | **0.72** | **0.34** | | | **-0.19** | **13.4** | **23.3** | **51.4** | **8.8** | **15.7** | | | **29.8** |
|  | |  | | | | | | | | | | | | | | | | | |

**Table 3.** Key Migration indicators: 1950/2000/2050

| COUNTRY | Net number of migrants, both sexes combined (thousands) | | | Net migration rate (per 1,000 population) | | |
| --- | --- | --- | --- | --- | --- | --- |
|  | 1950-1955 | 2000-2005 | 2050-2055 | 1950-1955 | 2000-2005 | 2050-2055 |
| Palestinian National Authority | -69 | -190 | -24 | -14.3 | -11.2 | -0.5 |
| Egypt | -50 | -68 | -205 | -0.5 | -0.2 | -0.3 |
| Jordan | 123 | -94 | -19 | 45.0 | -3.7 | -0.3 |
| Syrian Arab Republic | -70 | -380 | -47 | -3.8 | -4.4 | -0.3 |
| Izrael | 262 | 103 | 47 | 35.2 | 3.3 | 0.7 |
| Algeria | -310 | -205 | -47 | -6.6 | -1.3 | -0.2 |
| Libya | 0 | -12 | 19 | 0.0 | -0.4 | 0.5 |
| Morocco | 0 | -695 | -285 | 0.0 | -4.7 | -1.3 |
| Tunisia | -19 | -139 | -19 | -1.0 | -2.8 | -0.3 |
| Lebanon | 2 | 550 | -19 | 0.2 | 30.5 | -0.7 |
| **ENP South Arabs Weighted average** | **-72** | **-209** | **-138** | **-0.5** | **-1.2** | **-0.3** |
|  |  |  |  |  |  |  |
| Armenia | 50 | -144 | -24 | 6.8 | -9.5 | -1.8 |
| Azerbaijan | 10 | 11 | -32 | 0.6 | 0.3 | -0.6 |
| Georgia | 104 | -309 | -47 | 5.7 | -13.4 | -2.8 |
| Belarus | -335 | -6 | 10 | -8.6 | -0.1 | 0.2 |
| Republic of Moldova | 90 | -12 | -14 | 7.3 | -0.6 | -0.9 |
| Ukraine | 44 | -165 | -38 | 0.2 | -0.7 | -0.2 |
| **ENP East Slavs Weighted average** | **-3** | **-126** | **-30** | **-0.1** | **-1.6** | **-0.5** |
|  |  |  |  |  |  |  |
| Austria | -78 | 178 | 95 | -2.2 | 4.4 | 2.2 |
| Belgium | 59 | 248 | 114 | 1.4 | 4.8 | 1.8 |
| Bulgaria | -118 | -83 | - 48 | -3.2 | -2.1 | -1.9 |
| Croatia | 0 | -3 | -2 | 0.0 | -0.1 | -0.1 |
| Cyprus | -14 | 62 | 24 | -5.4 | 12.5 | 3.4 |
| Czech Republic | 60 | 47 | 57 | 1.3 | 0.9 | 1.2 |
| Denmark | -18 | 46 | 72 | -0.8 | 1.7 | 2.3 |
| Estonia | 37 | -18 | -5 | 6.5 | -2.7 | -0.9 |
| Finland | -33 | 32 | 55 | -1.6 | 1.2 | 1.9 |
| France | 298 | 739 | 380 | 1.4 | 2.5 | 1.1 |
| Germany | -42 | 1 | 713 | -0.1 | 0.0 | 1.9 |
| Greece | 19 | 114 | 48 | 0.5 | 2.1 | 1.0 |
| Hungary | 25 | 66 | 29 | 0.5 | 1.3 | 0.7 |
| Ireland | -146 | 200 | 47 | -10.1 | 9.9 | 1.6 |
| Italy | -204 | 1 624 | 475 | -0.9 | 5.6 | 1.7 |
| Latvia | 28 | -83 | 0 | 2.8 | -7.2 | 0.0 |
| Lithuania | -61 | -84 | 0 | -4.7 | -4.9 | 0.0 |
| Luxembourg | 6 | 14 | 19 | 3.8 | 6.5 | 4.6 |
| Malta | -29 | 6 | 2 | -18.7 | 2.9 | 0.9 |
| Netherlands | -95 | 145 | 104 | -1.8 | 1.8 | 1.2 |
| Poland | 0 | 138 | -36 | 0.0 | 0.2 | -0.2 |
| Portugal | -265 | 178 | 93 | -6.2 | 3.4 | 2.0 |
| Romania | -2 | -490 | -52 | 0.0 | -4.5 | -0.7 |
| Slovakia | 65 | 1 | 5 | 3.6 | 0.0 | 0.2 |
| Slovenia | -20 | 15 | 6 | -2.7 | 1.5 | 0.6 |
| Spain | -436 | 2 829 | 475 | -3.0 | 13.4 | 2.1 |
| Sweden | 52 | 142 | 174 | 1.5 | 3.2 | 2.9 |
| United Kingdom | -358 | 968 | 808 | -1.4 | 3.3 | 2.1 |
| **EU28 Weighted average** | **-91** | **693** | **394** | **-0.7** | **3.1** | **1.5** |
